# Association Between Change-of-Direction Performance and Landing Impact Characteristics During a Single-Leg Forward Drop Jump Landing Task in Junior Soccer Players

**DOI:** 10.3390/jfmk11030312

**Published:** 2026-08-10

**Authors:** Shinya Kishi, Yuuji Kimura, Takao Minami

**Affiliations:** 1 Department of Rehabilitation, Takarazuka University of Medical and Health Care, 2252 Nakanoshima, Wakayama City 640-8392, Japan; 2 Sumiya Orthopedic Hospital, 333 Yoshida, Wakayama City 640-8392, Japan; yuji6kimura@gmail.com (Y.K.); crddp728@gmail.com (T.M.)

**Keywords:** change-of-direction performance, youth soccer, vertical ground reaction force

## Abstract

**Background and Objectives:** Efficient landing shock attenuation is important for both athletic performance and lower-extremity injury prevention in youth soccer players. Although landing biomechanics and change-of-direction (COD) performance have been widely investigated, the relationship between field-based COD performance and landing impact characteristics remains unclear. This study aimed to examine the relationships between linear sprint performance, COD performance, and landing impact during a standardized single-leg forward drop jump landing task in junior soccer players. **Methods:** Thirty-six junior soccer players (22 boys and 14 girls; 10–12 years) participated in this cross-sectional study. Linear sprint performance and COD performance were assessed using a 30 m sprint test and a 30 m zigzag run test, respectively. Landing impact was evaluated during a standardized single-leg forward drop jump landing task using a force plate. Body weight-normalized peak vertical ground reaction force (peak vGRF, %BW) was calculated for each trial, and the average of the final five successful trials was used for analysis. Relationships between performance measures and body weight-normalized peak vGRF were examined using Spearman’s rank correlation coefficients and partial Spearman correlation analyses adjusted for sex. **Results:** No significant correlation was observed between 30 m sprint performance and body weight-normalized peak vGRF (ρ = 0.106, *p* = 0.537). In contrast, 30 m zigzag run performance showed a moderate positive correlation with body weight-normalized peak vGRF (ρ = 0.401, *p* = 0.015), indicating that poorer COD performance was associated with greater landing impact. This association remained significant after adjustment for sex (partial ρ = 0.474, *p* = 0.004). **Conclusions:** These findings suggest that landing impact characteristics may represent one biomechanical factor associated with COD performance. Although the observed association was moderate, field-based COD assessments may provide practical information regarding landing impact characteristics. Future studies should incorporate biological maturation, COD deficit, and additional biomechanical variables to further clarify these relationships.

## 1. Introduction

Soccer is a multidirectional sport characterized by repeated accelerations, decelerations, sprinting, cutting, jumping, and landing. Among these movements, change-of-direction (COD) ability is considered a fundamental determinant of successful performance because players are frequently required to rapidly decelerate and accelerate in response to unpredictable game situations. Efficient COD performance depends not only on muscular strength and sprinting ability but also on dynamic postural control, braking capacity, and neuromuscular coordination during high-intensity movements [1,2,3]. Consequently, understanding the biomechanical factors underlying COD performance is important for both athletic performance and lower-extremity injury prevention.

Landing is another essential movement in soccer, occurring repeatedly after jumping, heading, tackling, and other sport-specific actions. During landing, the lower extremities must absorb substantial impact forces while maintaining postural stability. Peak vertical ground reaction force (vGRF) is widely used to quantify external landing impact and characterize landing loading during functional tasks [4]. Higher peak vGRF reflects a greater magnitude of externally applied landing load, although it should be interpreted as an indicator of external loading rather than a direct measure of joint energy absorption [4]. Excessive landing impact has been associated with reduced hip and knee flexion, increased lower-extremity stiffness, and impaired neuromuscular control, all of which may increase mechanical loading of the lower extremities [4,5,6]. Therefore, assessment of landing biomechanics has become an important component of both performance evaluation and injury-related biomechanical assessment in young athletes.

Although sprinting and COD performance are often evaluated together in soccer, these physical capacities represent distinct movement skills. Linear sprint performance primarily reflects acceleration ability and maximal running speed, whereas COD performance additionally requires rapid braking, control of the body’s center of mass, eccentric muscle function, and subsequent re-acceleration [7]. Previous biomechanical studies have demonstrated that successful COD performance depends on effective braking mechanics, coordinated lower-extremity muscle activation, dynamic postural control, and efficient force production during directional transitions [8,9,10,11]. These biomechanical demands are comparable to those required during single-leg landing, where athletes must rapidly accommodate external impact forces while maintaining postural stability.

Both landing and COD movements therefore rely on efficient eccentric force absorption, coordinated neuromuscular activation, and precise control of the body’s center of mass. In contrast, although linear sprinting requires lower-extremity power and acceleration capacity, it involves substantially less braking and impact attenuation than multidirectional movements. Consequently, landing impact characteristics are theoretically more closely related to COD performance than to straight-line sprint performance.

Childhood and early adolescence represent critical periods for neuromuscular development. Growth and biological maturation influence muscle strength, coordination, balance, and movement efficiency, potentially affecting landing biomechanics and multidirectional movement performance [5,6,12]. Although biological maturation is recognized as an important factor influencing movement performance during childhood, maturity status (e.g., peak height velocity or Tanner stage) was not assessed in the present study. Therefore, the findings should be interpreted with consideration of possible developmental differences among participants.

Because movement patterns established during this developmental stage may influence future athletic performance and lower-extremity loading characteristics, identifying biomechanical characteristics associated with COD performance in junior athletes may contribute to more effective neuromuscular training and injury-prevention strategies [13,14].

Despite the biomechanical similarities between landing and COD movements, few studies have directly examined the relationship between landing impact and field-based COD performance in junior soccer players. Most previous investigations have evaluated landing biomechanics, COD performance, or maturation-related changes independently, and it remains unclear whether practical field-based performance tests reflect laboratory-derived measures of landing impact [5,8,9,12]. Clarifying this relationship may improve understanding of the biomechanical factors associated with multidirectional movement and provide clinicians, physical therapists, athletic trainers, and coaches with practical tools for identifying athletes who may benefit from targeted neuromuscular training.

Therefore, the purpose of this study was to investigate the relationships between linear sprint performance, COD performance, and landing impact measured during a standardized single-leg forward drop-jump landing task in junior soccer players. Based on the shared biomechanical and neuromuscular demands of landing and directional-change movements, we hypothesized that poorer COD performance would be associated with greater body weight-normalized landing impact. In contrast, because linear sprint performance primarily reflects acceleration and maximal running speed rather than braking and impact attenuation, we further hypothesized that sprint performance would demonstrate little or no association with landing impact.

## 2. Materials and Methods

### 2.1. Study Design

This cross-sectional observational study investigated the relationships between linear sprint performance, change-of-direction (COD) performance, and landing impact characteristics in junior soccer players. The study was conducted in accordance with the Strengthening the Reporting of Observational Studies in Epidemiology (STROBE) guidelines.

### 2.2. Participants

Thirty-six junior soccer players (22 boys and 14 girls; age range, 10–12 years) voluntarily participated in this study. All participants belonged to local community soccer clubs and regularly participated in organized soccer training at least twice per week.

Participants were eligible if they (1) were actively participating in soccer training, (2) were free from pain during testing, and (3) were able to complete all performance tests. Exclusion criteria included (1) any lower-extremity injury within the previous three months, (2) neurological or musculoskeletal disorders affecting athletic performance, and (3) inability to complete all measurements.

Written informed consent was obtained from all participants and their parents or legal guardians before participation.

### 2.3. Ethical Approval

This study was approved by the Ethics Committee of Social Medical Corporation Sumiya (Approval No. 2024-135) and was conducted in accordance with the principles of the Declaration of Helsinki.

### 2.4. Experimental Procedures

All measurements were completed during a single testing session conducted indoors under identical environmental conditions.

Before testing, participants performed a standardized warm-up consisting of approximately 10 min of light jogging, dynamic stretching, and running drills.

To minimize the influence of fatigue, performance tests were completed in the following order:

30 m sprint test;

30 m zigzag run test;

Single-leg forward drop jump landing task;

A rest period of approximately 2–3 min was provided between tests.

### 2.5. 30 m Sprint Test

Linear sprint performance was evaluated using a 30 m sprint test. Participants started from a standing position with the lead foot placed immediately behind the starting line. After the command “Ready,” an auditory start signal was given following an approximately 3 s pause. Participants were instructed to sprint maximally through the finish line without decelerating. Sprint time was recorded to the nearest 0.001 s using a wireless electronic timing gate system (Witty GATE, Microgate, Bolzano, Italy). Two successful trials were performed, and the faster time was used for subsequent statistical analyses.

### 2.6. 30 m Zigzag Run Test

**Figure 1:**
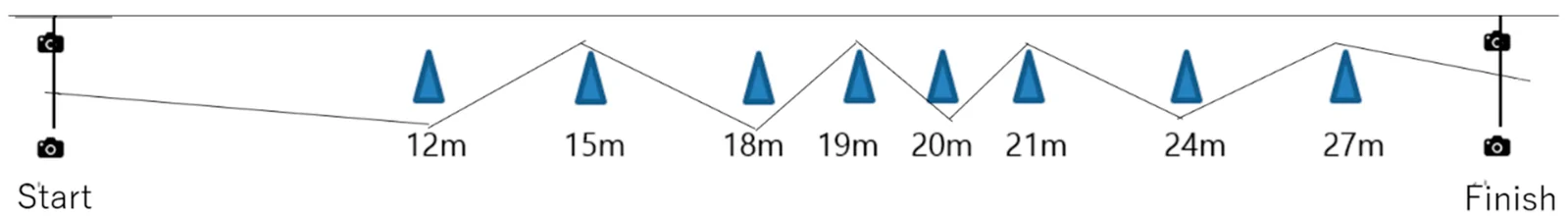
Configuration of the standardized 30 m zigzag run test.

Change-of-direction (COD) performance was evaluated using a standardized 30 m zigzag run test. The test course is illustrated in Figure 1. Eight cones (45 cm in height) were positioned at 12, 15, 18, 19, 20, 21, 24, and 27 m from the starting line. Participants were instructed to run through the course as quickly as possible while weaving alternately around the cones. The direction of entry around the first cone was left to the participant’s discretion. Running time was recorded to the nearest 0.001 s using a wireless electronic timing gate system (Witty GATE, Microgate, Bolzano, Italy). Two successful trials were performed, and the faster time was used for statistical analyses.

### 2.7. Single-Leg Forward Drop Jump Landing Task

Landing biomechanics were evaluated using a standardized single-leg forward drop jump landing task.

Participants stood on a 20 cm-high platform while balancing on the right leg. 

They were instructed to cross their arms over their chest and maintain this position throughout the task. 

Participants stepped forward from the platform without jumping and landed on the right lower extremity on the force plate positioned 30 cm in front of the platform.

Immediately after landing, participants maintained single-leg balance for 8 s while looking straight ahead.

The right lower extremity was evaluated in all participants to standardize testing procedures.

Before formal data collection, participants completed one to three familiarization trials according to individual performance. If the task was performed correctly on the first attempt, testing commenced immediately. Participants who required additional practice completed up to three familiarization trials.

Subsequently, ten successful trials were recorded.

Trials were repeated if participants uncrossed their arms, touched the contralateral foot to the floor, lost balance, or failed to maintain single-leg stance for the required duration.

### 2.8. Force Plate Measurements

Ground reaction force data were collected using a force plate system (SS-FP40AO-SY; Sports Sensing Co., Ltd., Fukuoka, Japan) at a sampling frequency of 1000 Hz. Before each testing session, the force plate was calibrated according to the manufacturer’s instructions to ensure accurate and reliable measurements.

Following the procedure described by Sugiyama et al. [15], participants performed ten successful landing trials, and the final five trials were used for analysis because landing performance was considered to be more stable during the latter half of repeated trials.

The primary biomechanical outcome of this study was the body weight-normalized peak vertical ground reaction force (peak vGRF, %BW). For each trial, peak vGRF was defined as the maximum vertical ground reaction force recorded immediately after initial foot contact during landing. The peak vGRF values obtained from the final five successful trials were averaged and used for statistical analyses.

**Figure 2:**
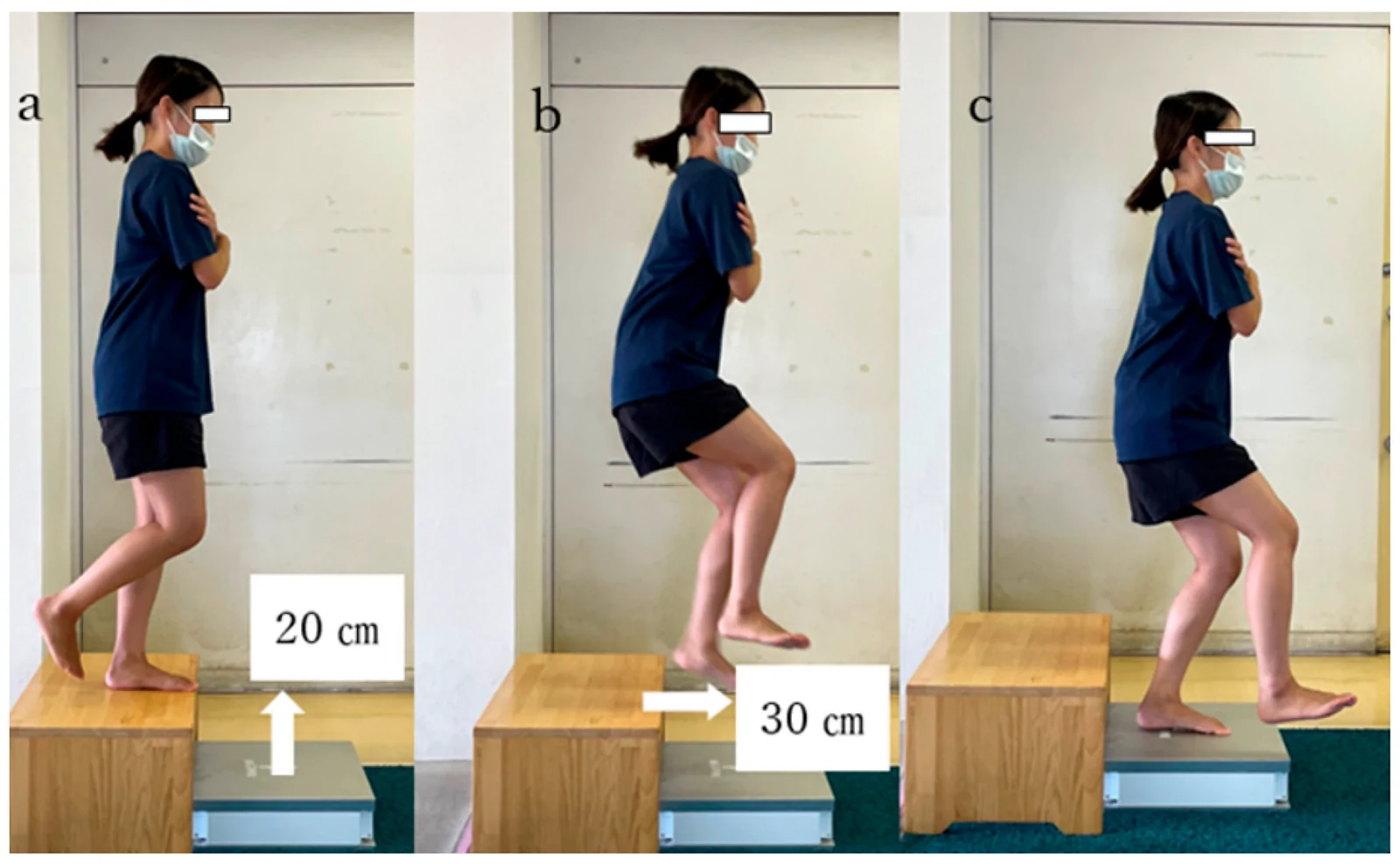
Single-leg forward drop jump landing task. (**a**): The participant stands on a 20 cm high platform placed behind the force plate, balancing on one leg. (**b**): The participant jumps forward onto the marked landing point on the force plate located 30 cm from the anterior edge of the platform. (**c**): After landing, the participant maintains a single-leg stance for 8 s.

To minimize the influence of body size, peak vGRF was normalized to each participant’s body weight and expressed as a percentage of body weight (%BW). A schematic illustration of the single-leg forward drop jump landing task is shown in Figure 2.

### 2.9. Statistical Analysis

All statistical analyses were performed using EZR version 1.68 (Saitama Medical Center, Jichi Medical University, Saitama, Japan), which provides a graphical user interface for R (The R Foundation for Statistical Computing, Vienna, Austria).

Data normality was assessed using the Shapiro–Wilk test. Because several variables did not satisfy the assumption of normality, comparisons between boys and girls were performed using the Mann–Whitney U test.

Because several variables did not satisfy the assumption of normality, relationships between performance measures and body weight-normalized peak vertical ground reaction force (peak vGRF, %BW) were assessed using Spearman’s rank correlation coefficients. 

To determine whether the observed relationships were independent of sex, partial Spearman correlation analyses adjusted for sex were additionally performed.

Correlation coefficients were interpreted as negligible (<0.20), weak (0.20–0.39), moderate (0.40–0.59), strong (0.60–0.79), and very strong (≥0.80).

All statistical tests were two-sided, and statistical significance was defined as *p* < 0.05.

## 3. Results

### 3.1. Participant Characteristics

Thirty-six junior soccer players, comprising 22 boys and 14 girls, completed all performance tests and were included in the final analyses. Participant characteristics are presented in Table 1. For the total sample, the median [interquartile range] values were 147.25 [142.75–151.03] cm for height, 36.50 [32.00–41.00] kg for body mass, 5.285 [5.212–5.465] s for the 30 m sprint time, 7.275 [7.079–7.457] s for the 30 m zigzag run time, and 473.33 [415.03–549.06] %BW for body weight-normalized peak vertical ground reaction force.

### 3.2. Comparisons Between Boys and Girls

Comparisons between boys and girls are presented in Table 2. Boys demonstrated significantly faster 30 m sprint times than girls (median [interquartile range]: 5.243 [5.186–5.417] s vs. 5.397 [5.260–5.482] s, respectively; *p* = 0.046).

No significant differences between boys and girls were observed in height (147.80 [144.38–152.75] cm vs. 146.35 [142.03–149.38] cm, respectively; *p* = 0.436), body mass (37.40 [33.03–41.00] kg vs. 33.50 [31.55–36.50] kg, respectively; *p* = 0.066), 30 m zigzag run time (7.222 [7.066–7.426] s vs. 7.380 [7.144–7.619] s, respectively; *p* = 0.224), or body weight-normalized peak vertical ground reaction force (487.40 [433.38–554.54] %BW vs. 442.83 [401.66–499.75] %BW, respectively; *p* = 0.168).

### 3.3. Relationships Between Field Performance and Landing Impact

Correlations between the field-based performance tests and body weight-normalized peak vertical ground reaction force are summarized in Table 3.

No significant correlation was observed between 30 m sprint time and body weight-normalized peak vertical ground reaction force (Spearman’s ρ = 0.106, 95% CI: −0.230 to 0.420, *p* = 0.537). This relationship remained non-significant after adjustment for sex (partial Spearman’s ρ = 0.204, 95% CI: −0.138 to 0.503, *p* = 0.239).

**Figure 3:**
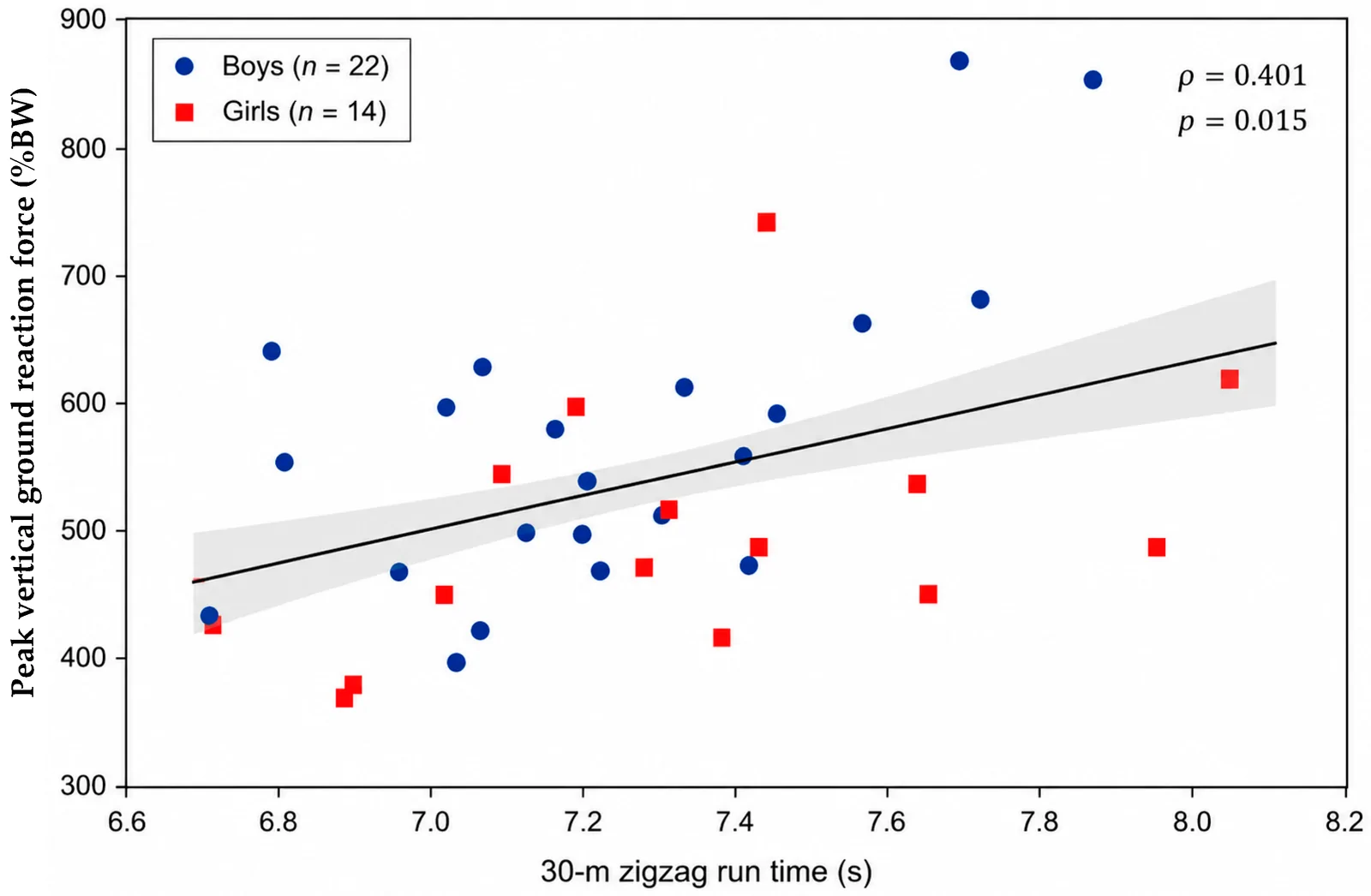
Relationship between 30 m zigzag run performance and body weight-normalized peak vertical ground reaction force (%BW) during the single-leg forward drop jump landing task.

In contrast, a significant positive correlation was observed between 30 m zigzag run time and body weight-normalized peak vertical ground reaction force (Spearman’s ρ = 0.401, 95% CI: 0.083 to 0.645, *p* = 0.015). This association remained significant after adjustment for sex (partial Spearman’s ρ = 0.474, 95% CI: 0.167 to 0.697, *p* = 0.004). Longer 30 m zigzag run times, indicating poorer change-of-direction performance, were associated with higher body weight-normalized peak vertical ground reaction forces. The relationship between 30 m zigzag run performance and body weight-normalized peak vertical ground reaction force is illustrated in Figure 3.

Eight cones (45 cm in height) were positioned at 12, 15, 18, 19, 20, 21, 24, and 27 m from the starting line. Participants ran through the course as quickly as possible while weaving alternately around the cones.

Scatter plot illustrating the relationship between 30 m zigzag run performance and body weight-normalized peak vertical ground reaction force (%BW) during the single-leg forward drop jump landing task in junior soccer players. Longer zigzag run times indicate poorer change-of-direction performance. Circles represent boys (*n* = 22), and squares represent girls (*n* = 14). The solid line represents the regression line, and the shaded area indicates the 95% confidence interval. A significant positive correlation was observed between zigzag run time and body weight-normalized peak vertical ground reaction force (Spearman’s ρ = 0.401, *p* = 0.015).

## 4. Discussion

The present study investigated the relationships between linear sprint performance, change-of-direction (COD) performance, and landing impact characteristics during a standardized single-leg forward drop-jump landing task in junior soccer players. The principal finding was that poorer COD performance was significantly associated with greater body weight-normalized landing impact, whereas no significant association was observed between linear sprint performance and landing impact. Furthermore, the relationship between COD performance and landing impact remained significant after adjustment for sex, suggesting that this association was largely independent of sex-related differences. These findings support our hypothesis and provide new evidence that field-based COD performance is associated with biomechanical characteristics of landing impact during single-leg landing tasks.

Soccer is characterized by repeated multidirectional movements requiring rapid deceleration, directional changes, and subsequent acceleration. Efficient execution of these movements depends on coordinated neuromuscular control and the ability to absorb and redistribute external forces through the lower extremities. Recent biomechanical studies have demonstrated that successful COD performance depends not only on muscular strength and sprint ability but also on effective braking mechanics, dynamic postural control, coordinated neuromuscular activation, and rapid force production during directional transitions [8,9,10]. The present findings extend these observations by demonstrating that athletes with poorer COD performance also exhibited greater body weight-normalized landing impact during a standardized single-leg forward drop-jump landing task. This finding suggests that landing impact characteristics and COD performance share common neuromuscular and biomechanical demands.

The relationship observed between COD performance and landing impact is biomechanically plausible. During both landing and directional-change movements, athletes must rapidly decelerate the body’s center of mass while maintaining lower-extremity alignment and postural stability. These movements require coordinated eccentric contractions of the hip extensors, knee extensors, plantar flexors, and trunk musculature to accommodate external loading efficiently. Recent evidence indicates that greater peak vertical ground reaction force reflects greater externally applied landing impact and is influenced by lower-extremity neuromuscular activation and landing strategy during single-leg landing tasks [4]. Furthermore, a recent systematic review demonstrated that landing biomechanics characterized by reduced lower-extremity flexion and altered neuromuscular control are associated with greater mechanical loading during landing tasks [5]. In addition, maturation has been shown to influence lower-extremity biomechanics during cutting and landing tasks in young soccer players, suggesting that developmental factors contribute to landing movement strategies [6]. Although injury occurrence was not investigated in the present study, our findings suggest that greater body weight-normalized landing impact may represent a biomechanical characteristic associated with less efficient movement control during multidirectional athletic tasks. Collectively, these findings support the concept that efficient impact attenuation and neuromuscular control are shared biomechanical characteristics underlying both landing and COD performance.

The present findings are also consistent with recent investigations of COD biomechanics. Recent systematic reviews have demonstrated that successful COD performance depends on effective braking mechanics, dynamic control of the body’s center of mass, coordinated lower-extremity muscle activation, and efficient force production during directional transitions [8,9]. Furthermore, faster COD performance has been associated with shorter ground-contact times, efficient braking and propulsive force production, and coordinated lower-extremity joint mechanics [8,9,10]. These biomechanical characteristics closely resemble those required during single-leg landing, supporting the present findings that poorer COD performance is associated with greater landing impact. Condello et al. further demonstrated that biomechanical strategies adopted during COD tasks influence movement efficiency and mechanical loading [16]. Likewise, increased movement speed has been shown to substantially alter lower-extremity mechanical loading during side-cutting maneuvers [17]. Collectively, these findings further support the concept that effective force attenuation and neuromuscular control are essential determinants of successful COD performance. The present study complements these previous findings by demonstrating that a simple field-based COD test is associated with force plate-derived landing impact characteristics in junior soccer players.

The relationship between COD performance and landing impact remained significant after adjustment for sex. Growth and biological maturation substantially influence muscle strength, coordination, balance, and movement efficiency during childhood and adolescence [5,6,14]. Recent evidence indicates that maturation influences lower-extremity biomechanics during landing and change-of-direction tasks, although these effects may vary according to sex, task characteristics, and the biomechanical variables evaluated [5,6]. Furthermore, recent findings in adolescent male soccer players suggest that maturity status is associated with jump-performance characteristics, whereas maturity-related differences in landing-force variables may not always be evident [12]. Although biological maturity was not assessed in the present study, the significant association between landing impact and COD performance persisted after adjustment for sex, suggesting that this relationship is not explained solely by sex-related differences. Nevertheless, residual developmental differences among participants should be considered when interpreting the present findings.

The present findings have important practical implications for clinicians, physical therapists, athletic trainers, strength and conditioning coaches, and youth soccer coaches. Field-based COD tests are inexpensive, require minimal equipment, and can be easily incorporated into routine physical performance assessments. The present results suggest that these practical assessments may also provide indirect information regarding movement quality associated with landing impact characteristics. Consequently, combining field-based COD assessments with force plate evaluation, when available, may improve the identification of young athletes who demonstrate greater landing impact during functional tasks and who may benefit from individualized neuromuscular training programs.

Recent intervention studies support this interpretation. Neuromuscular training programs targeting movement quality have been shown to improve landing biomechanics and functional movement competency in youth athletes [18,19]. Furthermore, appropriately supervised strength and neuromuscular training during childhood and adolescence improves muscular strength, movement competency, and athletic performance without increasing injury risk [20]. Recent systematic reviews further demonstrate that resistance training and combined neuromuscular training effectively improve COD performance in youth athletes [14], while strength, plyometric, and combined training enhance strength, power, speed, and multidirectional performance in highly trained male youth soccer players [21]. Collectively, these findings support the clinical relevance of the present results and suggest that interventions targeting landing impact characteristics and neuromuscular control may contribute to improved COD performance and safer movement strategies during sports participation.

The relationship identified in the present study may also be interpreted from the perspective of long-term athletic development. Childhood and early adolescence represent critical periods for developing movement competency, coordination, balance, and neuromuscular function that provide the foundation for future athletic performance [21]. Because both landing biomechanics and COD performance continue to develop throughout growth and maturation, identifying movement deficits during this developmental period may facilitate individualized training programs aimed at optimizing athletic development while potentially reducing excessive lower-extremity loading. Therefore, assessment of landing impact together with practical field-based COD testing may represent a useful strategy for monitoring movement performance in junior soccer players.

The present study has several strengths. First, landing impact was quantified objectively using a force plate during a standardized single-leg forward drop-jump landing task, providing reliable biomechanical measurements under controlled conditions. Second, laboratory-based biomechanical assessment was combined with practical field-based performance tests that are readily applicable in youth sports settings, thereby enhancing the clinical relevance of the findings. Third, vertical ground reaction force was normalized to body weight, thereby minimizing the influence of body size differences among growing children and improving comparability among participants. Finally, the significant association between COD performance and landing impact remained after adjustment for sex, suggesting that the observed relationship was not explained solely by sex-related differences.

Several limitations should be acknowledged. First, because of the cross-sectional study design, causal relationships cannot be established. Although greater body weight-normalized landing impact was associated with poorer COD performance, it remains unclear whether impaired landing impact attenuation contributes directly to reduced COD ability or whether both variables reflect common neuromuscular characteristics. Second, the relatively small sample size and recruitment of participants from local youth soccer clubs may limit the generalizability of the findings. Third, biological maturation, lower-extremity muscle strength, joint kinematics, muscle activation patterns, and training history were not assessed. These factors are known to influence both landing biomechanics and multidirectional movement performance during growth and maturation and should therefore be incorporated into future investigations [5,6,12]. Finally, the present study focused on body weight-normalized peak vertical ground reaction force during landing. Future studies should include additional biomechanical variables, such as loading rate, lower-extremity joint kinematics, center-of-pressure characteristics, and electromyographic activity, to further clarify the mechanisms underlying COD performance.

The present study directly addresses the research gap identified in the Introduction. Previous investigations have independently examined landing biomechanics and COD performance; however, evidence linking these functional characteristics in junior soccer players has remained limited. To the best of our knowledge, this is the first study to demonstrate that body weight-normalized landing impact measured during a standardized single-leg forward drop-jump landing task is significantly associated with field-based COD performance in junior soccer players, whereas no significant relationship was observed with linear sprint performance. These findings provide novel evidence that field-based COD performance is associated with landing impact characteristics in junior soccer players and highlight the potential value of simple field-based assessments for evaluating landing biomechanics and informing evidence-based performance enhancement and injury-prevention strategies.

## 5. Conclusions

Poorer change-of-direction performance was significantly associated with greater body weight-normalized landing impact during a standardized single-leg forward drop jump landing task in junior soccer players, whereas linear sprint performance showed no significant association with landing impact. Furthermore, the association between change-of-direction performance and landing impact remained significant after adjustment for sex, suggesting that this relationship is largely independent of sex-related differences. These findings suggest that efficient landing shock attenuation may represent an important biomechanical characteristic associated with change-of-direction performance in junior soccer players. Field-based change-of-direction assessments may therefore provide a practical approach for evaluating landing impact characteristics and identifying athletes who may benefit from targeted neuromuscular training aimed at improving multidirectional performance and reducing lower-extremity mechanical loading.

## Figures and Tables

**Table 1:** Participant Characteristics.

Variable	Total (*n* = 36)	Boys (*n* = 22)	Girls (*n* = 14)
Height (cm)	147.25 [142.75–151.03]	147.80 [144.38–152.75]	146.35 [142.03–149.38]
Body Mass (kg)	36.50 [32.00–41.00]	37.40 [33.03–41.00]	33.50 [31.55–36.50]
30 m Sprint Time (s)	5.285 [5.212–5.465]	5.243 [5.186–5.417]	5.397 [5.260–5.482]
30 m Zigzag Run Time (s)	7.275 [7.079–7.457]	7.222 [7.066–7.426]	7.380 [7.144–7.619]
Peak vGRF (%BW)	473.33 [415.03–549.06]	487.40 [433.38–554.54]	442.83 [401.66–499.75]

Values are presented as median [interquartile range]. Abbreviations: vGRF, vertical ground reaction force; %BW, percentage of body weight.

**Table 2:** Comparisons of participant characteristics between boys and girls.

Variable	Boys (*n* = 22)	Girls (*n* = 14)	*p* Value
Height (cm)	147.80 [144.38–152.75]	146.35 [142.03–149.38]	0.436
Body Mass (kg)	37.40 [33.03–41.00]	33.50 [31.55–36.50]	0.066
30 m Sprint Time (s)	5.243 [5.186–5.417]	5.397 [5.260–5.482]	0.046
30 m Zigzag Run Time (s)	7.222 [7.066–7.426]	7.380 [7.144–7.619]	0.224
Peak vGRF (%BW)	487.40 [433.38–554.54]	442.83 [401.66–499.75]	0.168

Values are presented as median [interquartile range]. Comparisons between boys and girls were performed using the Mann–Whitney U test. Abbreviations: vGRF, vertical ground reaction force; %BW, percentage of body weight.

**Table 3:** Correlations between field performance tests and body weight-normalized peak vertical ground reaction force (vGRF, %BW).

Variable	Spearman rho	95% CI	*p* Value	Partial rho (Sex-Adjusted)	95% CI	*p* Value
30-m Sprint Time	0.106	−0.230 to 0.420	0.537	0.204	−0.138 to 0.503	0.239
30-m Zigzag Run Time	0.401	0.083 to 0.645	0.015	0.474	0.167 to 0.697	0.004

Abbreviations: vGRF, vertical ground reaction force; CI, confidence interval; %BW, percentage of body weight. Partial Spearman correlation coefficients were adjusted for sex.

## Data Availability

The data presented in this study are available from the corresponding author upon reasonable request. The data are not publicly available because they contain information that could compromise the privacy of the participants.
